# Supplementary material for: Cohort Study of Maternal Gestational Weight Gain, Gestational Diabetes, and Childhood Asthma
Source: Nutrients. 2022 Dec 6;14(23):5188. doi: 10.3390/nu14235188 (PMC9741125; doi:10.3390/nu14235188)
Supplement: Supplementary file 1 [file nutrients-14-05188-s001.zip › nutrients-2043537-supplementary.pdf]

Prospective study of maternal gestational weight gain, gestational diabetes and childhood asthma

Supplementary materials

Orianne Dumas, Anna Chen Arroyo, M. Kamal Faridi, Kaitlyn James, Sarah Hsu, Camille Powe, Carlos A. Camargo Jr.

Table S1. Comparison of maternal and child's characteristics in mother-child pairs with or without missing data for GDM.

	Missing data for GDM		
	No (n=15,171)	Yes (n=1180)	P
**Maternal characteristics**			
Early pregnancy BMI, kg/m2, %			<0.001
< 20.0	8.4	5.7	
20.0-22.4	19.9	16.3	
22.5-24.9	22.9	18.4	
25-29.9	29.0	27.1	
≥30	19.9	32.5	
GWG, lb, %			<0.001
<15	12.7	28.8	
15-24	25.3	26.2	
25-34	34.4	27.8	
35-44	19.9	12.3	
≥45	7.8	4.9	
GWG relative to recommendations, %			<0.001
Under recommended weight gain	22.9	36.2	
Meets recommended weight gain	36.6	32.9	
Over recommended weight gain	40.5	30.9	
C-section, %	27.8	35.1	<0.001
Nulliparous, %			<0.001
Yes	47.7	42.3	
No	49.3	53.6	
Missing	3.0	4.1	
Age at delivery, mean (SD)	30.3 (6.3)	30.4 (6.8)	0.72
Maternal Race/ethnicity, %			0.008
White	51.5	47.2	
Black	7.5	9.7	
Hispanic	20.0	22.3	
Asian	7.6	7.3	
Other	13.4	13.6	
Maternal asthma, %	8.0	8.1	0.95
Smoking status (3 months prior to pregnancy or during pregnancy), %	7.6	10.1	0.002
Insurance status at birth, %			<0.001
Private	55.5	48.6	

Table S1. Comparison of maternal and child's characteristics in mother-child pairs with or without missing data for GDM.

	Missing data for GDM		
	No (n=15,171)	Yes (n=1180)	P
Public	34.3	40.5	
Limited	6.5	6.8	
Other	3.6	4.1	
**Child's characteristics**			
Female, %	47.8	48.3	0.76
Birth weight, lb, %			<0.001
<5.5	6.4	13.6	
5.5-6.9	26.8	24.8	
7.0-8.4	49.3	42.5	
8.5-9.9	16.1	15.4	
≥10	1.4	3.7	
Gestational age, %			<0.001
<32	0.67	6.1	
32-36	5.8	8.2	
≥37	93.5	85.7	
Atopic dermatitis, %	1426 (9.4)	8.5	0.29
Allergic rhinitis, %	1588 (10.5)	10.1	0.68

BMI – Body Mass Index; GDM - gestational diabetes mellitus; GWG – Gestational Weight Gain
% missing values is displayed for variables with >3% missing values
Women with missing data for GDM include women known to have pre-existing diabetes, who were not screened for GDM, and women with abnormal GLT and no / incomplete OGTT

Table S2. Associations of maternal BMI and GWG with incidence of asthma in childhood according to the child's sex

	OR (95% CI) - boys	OR (95% CI) - girls	p-inter
Maternal early pregnancy BMI, kg/m^2			
< 20.0	1.20 (0.94-1.53)	0.90 (0.65-1.25)	0.11
20.0-22.4 (ref.)	1	1	
22.5-24.9	**1.22 (1.01-1.47)**	1.07 (0.85-1.35)	0.45
25-29.9	1.13 (0.94-1.36)	**1.31 (1.06-1.62)**	0.25
≥30	**1.33 (1.10-1.62)**	**1.26 (1.00-1.58)**	0.88
Maternal GWG (lb)†			
<15	1.03 (0.85-1.24)	1.09 (0.87-1.36)	0.57
15-24	1.01 (0.87-1.19)	0.88 (0.73-1.06)	0.25
25-34 (ref.)	1	1	
35-44	0.97 (0.81-1.14)	0.97 (0.79-1.19)	0.98
≥45	1.12 (0.89-1.40)	0.84 (0.62-1.13)	0.17
GWG relative to recommendations†			
Under recommendations	1.05 (0.9-1.22)	1.05 (0.87-1.26)	0.82
Meets recommendations	1	1	
Over recommendations	0.99 (0.86-1.14)	1.03 (0.87-1.21)	0.44

BMI – Body Mass Index; GWG – Gestational Weight Gain; OR – Odds Ratio; CI – Confidence Interval. Analyses were adjusted for maternal age at delivery, asthma, maternal race/ethnicity, smoking status, insurance status at birth, mode of delivery (C-section). †Analyses were further adjusted for maternal early pregnancy BMI.

Table S3. Associations of GWG with incidence of asthma in childhood according to maternal early pregnancy BMI.

Maternal GWG (lb)	OR (95% CI) - 20-24.9 kg/m^2	OR (95% CI) - ≥25 kg/m^2	p-inter
<15	0.97 (0.69-1.35)	0.96 (0.80-1.16)	0.91
15-24	0.98 (0.80-1.18)	0.87 (0.73-1.04)	0.37
25-34 (ref.)	1	1	-
35-44	0.88 (0.72-1.07)	1.03 (0.85-1.26)	0.26
≥45	0.98 (0.74-1.30)	1.02 (0.78-1.32)	0.79
GWG relative to recommendations			
Under recommendations	0.99 (0.82-1.18)	1.04 (0.85-1.27)	0.76
Meets recommendations	1	1	-
Over recommendations	0.92 (0.77-1.11)	1.10 (0.95-1.28)	0.12

BMI – Body Mass Index; GWG – Gestational Weight Gain; OR – Odds Ratio; CI – Confidence Interval. Analyses were adjusted for maternal age at delivery, asthma, maternal race/ethnicity, smoking status, insurance status at birth, mode of delivery (C-section).

Table S4. Associations of GDM with incidence of allergic and non-allergic asthma in childhood, with further adjustment for gestational age.

	All asthma		Non-allergic asthma		Allergic asthma	
	No of cases	OR (95% CI)	No of cases	OR (95% CI)	No of cases	OR (95% CI)
GDM, Carpenter-Coustan criteria						
No	1,980	1	1,301	1	679	1
Yes	122	1.17 (0.95-1.45)	78	1.11 (0.86-1.44)	44	1.25 (0.91-1.74)
GDM, National Diabetes Data Group criteria						
No	2,015	1	1,318	1	697	1
Yes	87	**1.37 (1.06-1.76)**	61	**1.44 (1.07-1.94)**	26	1.20 (0.79-1.82)

BMI – Body Mass Index; GDM – gestational diabetes mellitus; OR – Odds Ratio; CI – Confidence Interval. Analyses were adjusted for maternal early pregnancy BMI, age at delivery, asthma, maternal race/ethnicity, smoking status, insurance status at birth, mode of delivery (C-section), and gestational age.

Table S5. Associations of GDM with incidence of asthma in childhood according to the child's sex.

	OR (95% CI) - boys	OR (95% CI) - girls	p-inter
GDM, Carpenter-Coustan criteria			
No	1	1	
Yes	1.20 (0.92-1.57)	1.26 (0.91-1.74)	0.56
GDM, National Diabetes Data Group criteria			
No	1	1	
Yes	**1.54 (1.12-2.12)**	1.28 (0.85-1.93)	0.70

BMI – Body Mass Index; GDM – gestational diabetes mellitus; OR – Odds Ratio; CI – Confidence Interval. Analyses were adjusted for maternal early pregnancy BMI, age at delivery, asthma, maternal race/ethnicity, smoking status, insurance status at birth, mode of delivery (C-section).
